# Supplementary figures and images for: morphoHeart: A quantitative tool for integrated 3D morphometric analyses of heart and ECM during embryonic development
Source: PLoS Biol. 2025 Jan 29;23(1):e3002995. doi: 10.1371/journal.pbio.3002995 (PMC11778784; doi:10.1371/journal.pbio.3002995)

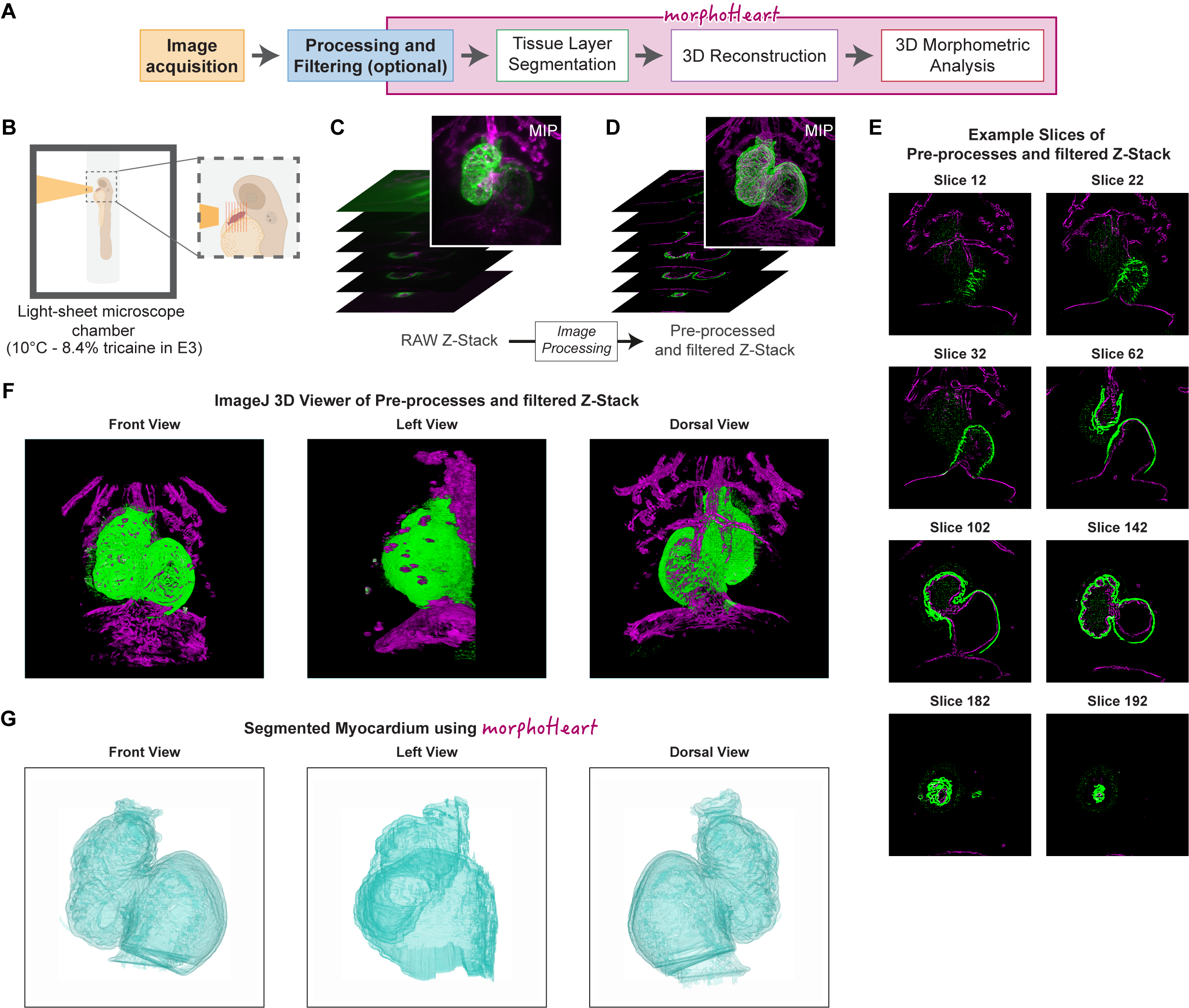

Supplement: S1 Fig — (A) Schematic overview of morphoHeart’s pipeline. (B–D) Overview of the sample acquisition and preparation for the data presented in this manuscript. The hearts of zebrafish larvae were temporarily arrested in a lightsheet chamber (B), and z-stack images acquired (C) which were preprocessed prior to use in morphoHeart (D). E: Example slices at progressive z-planes through a processed and filtered z-stack of a 72hpf Tg(myl7:lifeACT:GFP);Tg(fli1a:AC-TagRFP) heart, with the myocardial actin labelled in green and endothelial actin labelled in magenta. (F) Snapshots of 3D renderings of processed and filtered 72–74 hpf hearts using ImageJ’s 3D Viewer from a ventral, left, and dorsal view. (G) Snapshots of the 3D segmented heart (same hearts as shown in F) using morphoHeart from a ventral, left, and dorsal view. (TIF) [file pbio.3002995.s001.tif]

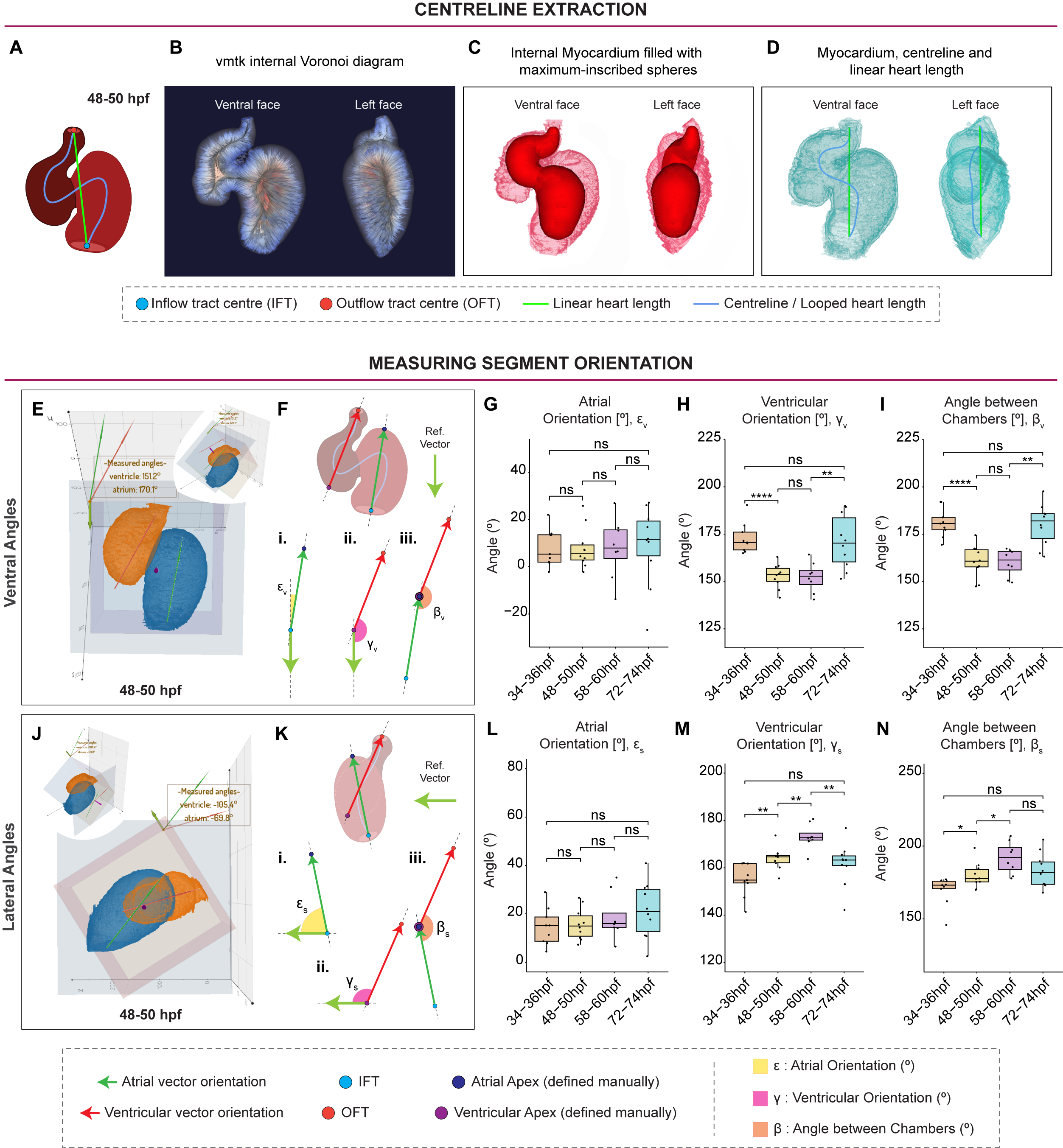

Supplement: S2 Fig — (A) Schematics depicting both linear (green) and looped (blue) lines through the heart between inflow and outflow poles. The blue looped line represents the “centreline” through the tissue generated by assuming the heart as a tubular structure. (B) Internal Voronoi diagram generated when extracting the centreline using the vmtk package integrated into morphoHeart. (C) Internal myocardial mesh filled with maximum-inscribed spheres used to calculate each point of the centreline. (D) Myocardial tissue meshes showing linear heart length (green, linear distance between poles) and looped heart length (blue, vmtk-calculated centreline through tissue between poles). (E–N) Heart morphogenesis is accompanied by relative chamber realignments. Screenshot depicting morphoHeart’s method for measuring chamber orientations from the heart’s ventral face (E), and schematic depicting how each chamber angle is calculated with respect to a reference vector (F). The atrium remains static (G), while the ventricle first moves substantially clockwise, and after looping pivots counter-clockwise (H), resulting in a frontal displacement and realignment of the chambers as the heart loops, grows, and compacts (I). Screenshot depicting morphoHeart’s method for measuring lateral chamber orientations (J), and schematic depicting how each chamber lateral angle is calculated with respect to a reference vector (K). Each chamber angle is measured, and the lateral angle between them calculated (N). The atrium’s lateral position remains relatively unchanged (L), while the ventricle straightens as the heart loops, and becomes laterally displaced as it compacts (M). This results in repositioning of the chambers or lateral rotation around the AVC (N). One-way ANOVA with multiple comparisons. * p < 0.05, ** p < 0.01, *** p < 0.001, **** p < 0.0001; 34–36 hpf: n = 9; 48–50 hpf: n = 10; 58–60 hpf: n = 8; 72–74 hpf: n = 10. Plots display median and quartiles. The numerical data underlying this figure can be fou [file pbio.3002995.s002.tif]

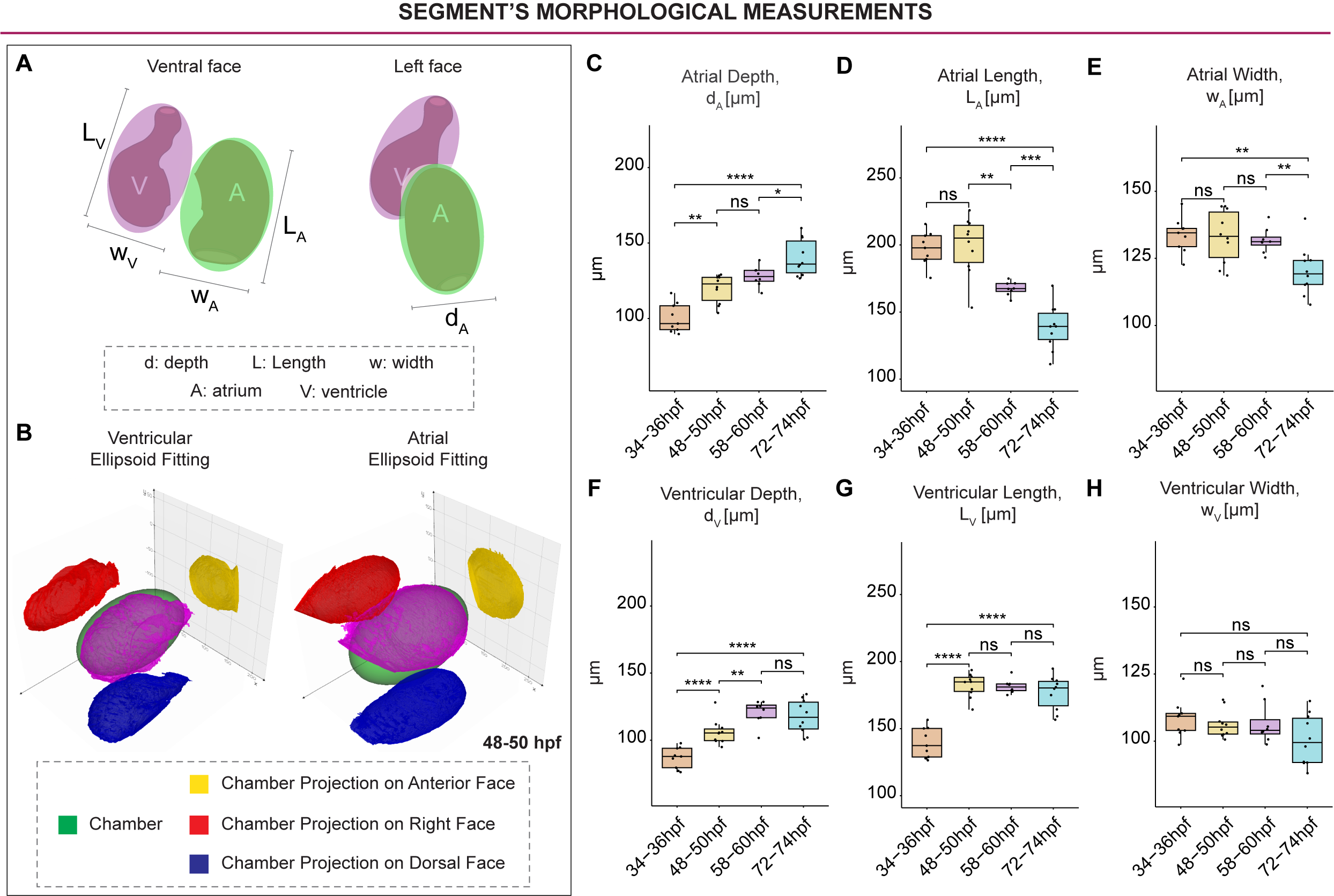

Supplement: S3 Fig — (A) Illustration of the geometrical measurements acquired for both atrium (A) and ventricle (V), including chamber depth (d), length (L) and width (w). (B) Individual chamber meshes (magenta) are projected to the anterior (yellow), right (red), and dorsal (blue) face of a reference organ cube, and its largest dimensions are used to create an ellipsoid that best resembles its shape (green). (C–H) Quantification of chamber depth (C, F), length (D, G), and width (E, H). The atrium expands in the z-plane, becoming deeper throughout development (C). Between 48 hpf and 74 hpf it also shortens (D) and narrows (E), becoming more spherical. The ventricle also expands in depth (F), while it lengthens (G) between 34 hpf and 50 hpf to become a more elongated shape. One-way ANOVA with multiple comparisons. * p < 0.5, ** p < 0.01, *** p < 0.001, **** p < 0.0001; 34–36 hpf: n = 9; 48–50 hpf: n = 10; 58–60 hpf: n = 8; 72–74 hpf: n = 10. Plots display median and quartiles. The numerical data underlying this figure can be found in S1 Data. (TIF) [file pbio.3002995.s003.tif]

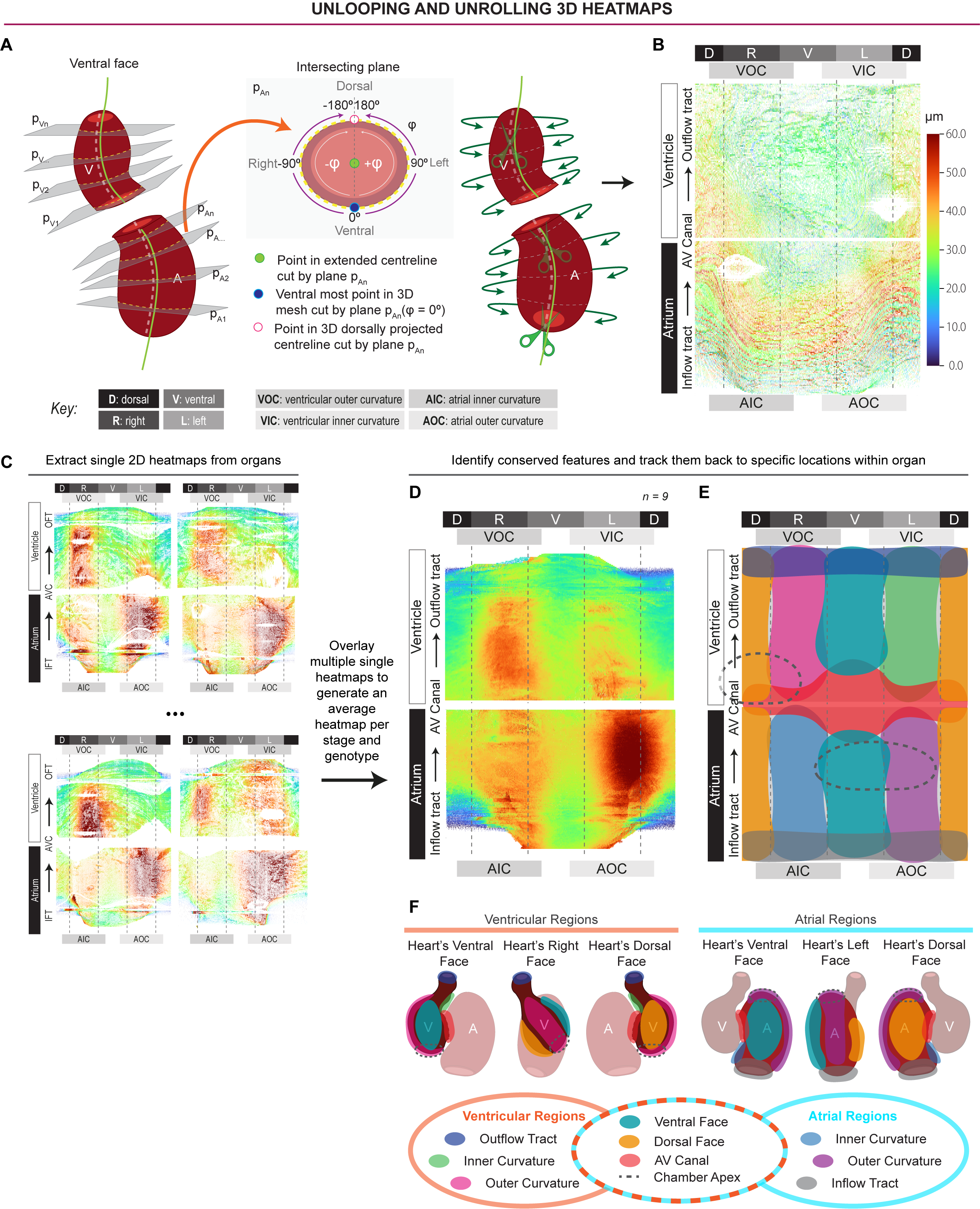

Supplement: S4 Fig — (A) Schematic depicting the method for unrolling 3D heatmaps into 2D heatmaps. The centreline (green) is extended beyond the poles of the heart, and a defined number of planes are cut through the heart mesh, transverse to the orientation of the centreline at each plane. For each intersecting plane, the ventral-most point of the mesh is cut and assigned position 0°, while the dorsal-most point of the mesh is cut and assigned position 180°/−180°, giving each mesh point in each plane a universal coordinate. As each mesh point is additionally associated to a measured thickness/ballooning value, together this allows the tube to be “unrolled” and mapped onto a standard 2D geometry (B). (C, D) As each heatmap has the same coordinate system, the thickness/ballooning measurement of multiple organs (at the same stage and of the same genotype; C) can be averaged at each coordinate, allowing individual heatmaps from multiple embryos to be combined, producing an average heatmap which represents typical phenotype independent of small biological variation in tissue morphology (D). (E, F) Schematics to facilitate interpretation of the heatmaps, demonstrating correspondence of the regions identified in the 2D heatmaps (E) and the 3D morphology of the organ (F), identified with different colours for each chamber. Labels around the heatmap indicate cardiac region: D—dorsal, V—ventral, L—left, R—right, AOC—atrial outer curvature, AIC—atrial inner curvature, VOC—ventricular outer curvature, VIC—ventricular inner curvature, AV Canal—atrioventricular canal. (TIF) [file pbio.3002995.s004.tif]

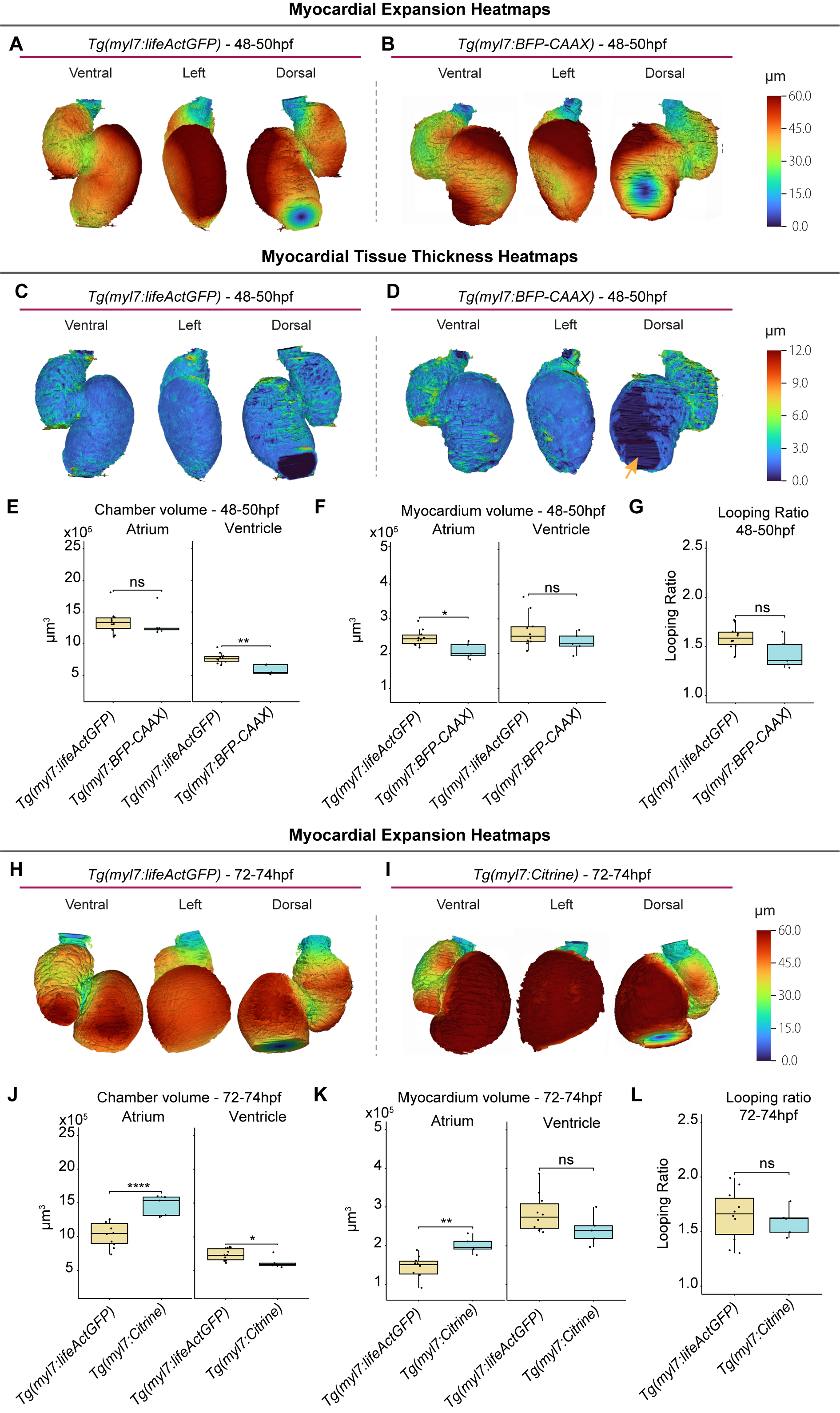

Supplement: S5 Fig — (A, B) 3D heatmap analysis of chamber (myocardial) ballooning in Tg(myl7:lifeActGFP) transgenic hearts (A) and Tg(myl7:BFP-CAAX) transgenic hearts (B) at 48–50 hpf. (C, D) 3D heatmap analysis of myocardial thickness in Tg(myl7:lifeActGFP) transgenic hearts (C) and Tg(myl7:BFP-CAAX) transgenic hearts (D) at 48–50 hpf. Parts of the dorsal atrial myocardium are missing in Tg(myl7:BFP-CAAX) transgenic embryos (yellow arrow, D). (E) Quantitative analysis of chamber volume reveals that ventricular chamber volume is smaller in Tg(myl7:BFP-CAAX) hearts compared to Tg(myl7:lifeAct-GFP) transgenics. (F) Quantitative analysis of myocardial volume reveals that atrial myocardium volume is smaller in Tg(myl7:BFP-CAAX) hearts compared to Tg(myl7:lifeAct-GFP) transgenics. (G) Analysis of looping ratio revealing that looping geometry is not significantly different between transgenes. (H, I) 3D heatmap analysis of chamber (myocardial) ballooning in Tg(myl7:lifeActGFP) transgenic hearts (H) and Tg(myl7:Citrine) transgenic hearts (I) at 72–74 hpf. (J) Quantitative analysis of chamber volume reveals that atrial volume is larger and ventricular chamber volume smaller in Tg(myl7:Citrine) hearts compared to Tg(myl7:lifeAct-GFP) transgenics. (K) Quantitative analysis of myocardial volume reveals that atrial myocardium is increased in Tg(myl7:Citrine) hearts compared to Tg(myl7:lifeAct-GFP) transgenics. (L) Analysis of looping ratio revealing that looping geometry is not significantly different between transgenes. One-way ANOVA with multiple comparisons. * p < 0.5, ** p < 0.01, *** p < 0.001, **** p < 0.0001. Tg(myl7:BFP-CAAX) 48 hpf n = 5; Tg(myl7:lifeActGFP) 48 hpf n = 10; Tg(myl7:Citrine) 72 hpf n = 5; Tg(myl7:lifeActGFP) n = 10. Plots display median and quartiles. The numerical data underlying this figure can be found in S1 Data. (TIF) [file pbio.3002995.s005.tif]

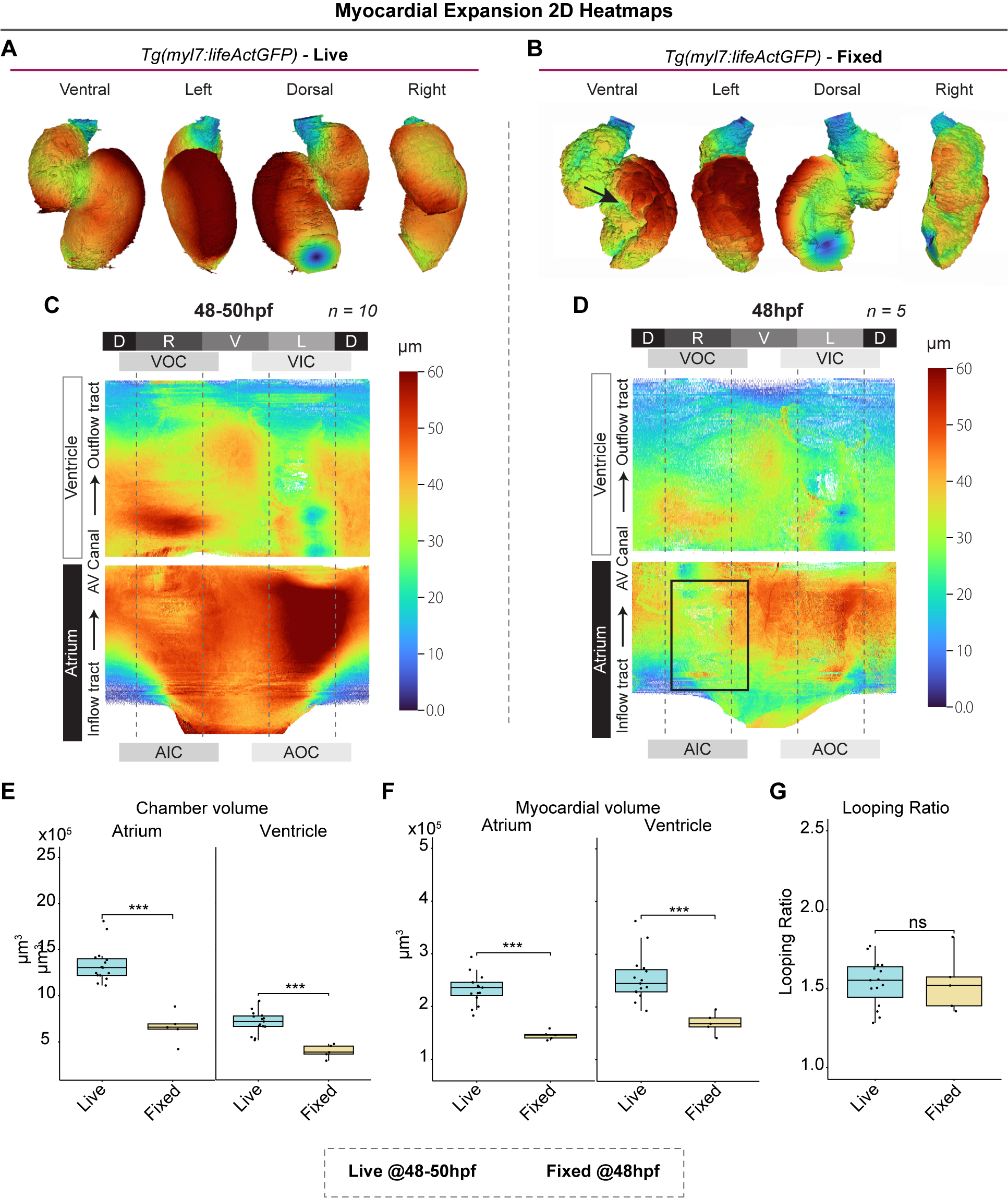

Supplement: S6 Fig — (A, B) 3D heatmap analysis of chamber (myocardial) ballooning in live (A) and fixed (B) Tg(myl7:lifeActGFP) transgenic embryos at 48–50 hpf. Arrow in (B) depicts folding of the atrial tissue in fixed samples that is not observed in live hearts. (C, D) Averaged 2D unrolled chamber ballooning heatmaps in live (C) and fixed (D) embryos, highlighting the reduction in chamber ballooning in fixed tissue. (E) Quantitative analysis of chamber volume reveals that both chambers in fixed hearts are smaller than live counterparts. (F) Quantitative analysis of myocardial volume demonstrates that tissue shrinkage occurs in fixed samples. (G) Analysis of looping ratio revealing that looping geometry is not significantly affected by fixation. One-way ANOVA with multiple comparisons. *** p < 0.001. Tg(myl7:lifeActGFP) 48 hpf live n = 10; Tg(myl7:lifeActGFP) 48 hpf fixed n = 5. Plots display median and quartiles. The numerical data underlying this figure can be found in S1 Data. (TIF) [file pbio.3002995.s006.tif]

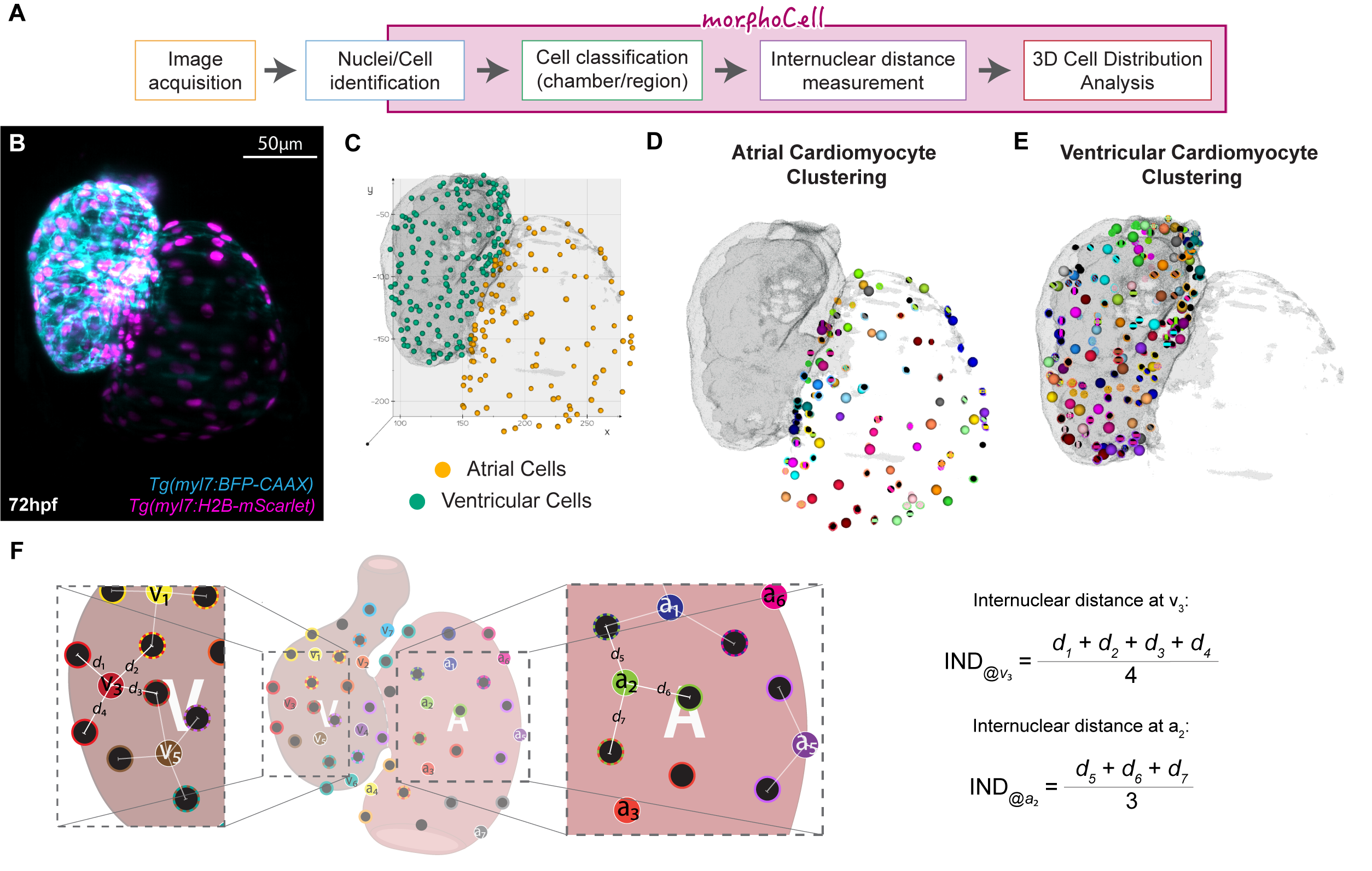

Supplement: S7 Fig — (A) Flow diagram describing the phases involved in the process of acquiring comprehensive cell data using morphoCell. Nuclei identification is performed using Imaris spot-finder function and given as input into morphoCell with the original z-stack images. (B) Maximum intensity projection of the heart of a Tg(myl7:BFP-CAAX); Tg(myl7:H2B-mScarlet) transgenic embryo, highlighting the myocardium (blue) and myocardial nuclei (magenta). (C–E) morphoCell renderings of myocardium and cardiomyocyte nuclei (spheres). Nuclei are initially not categorised into chambers; however, chambers can be separated via a user-defined plane, and nuclei subsequently automatically categorised as atrial (C; orange spheres) or ventricular (C; green spheres). (D–F) Cells identified in each chamber (atrium, ventricle) are clustered into distinct groups containing “seed” cells (single colour) or “neighbouring” cells (striped) that are representative of cell organisation within the chamber (D: atrial cluster, comprised of a central seed cell and 3 neighbouring cells, E: ventricular clusters, comprised of a central seed cell and 4 neighbouring cells). Seeds and neighbouring cells can be categorised to regions of interest within the chamber. The 3D distances between the seed cell and its neighbouring cells are measured and averaged to generate a single average internuclear distance value for each seed (F). (TIF) [file pbio.3002995.s007.tif]

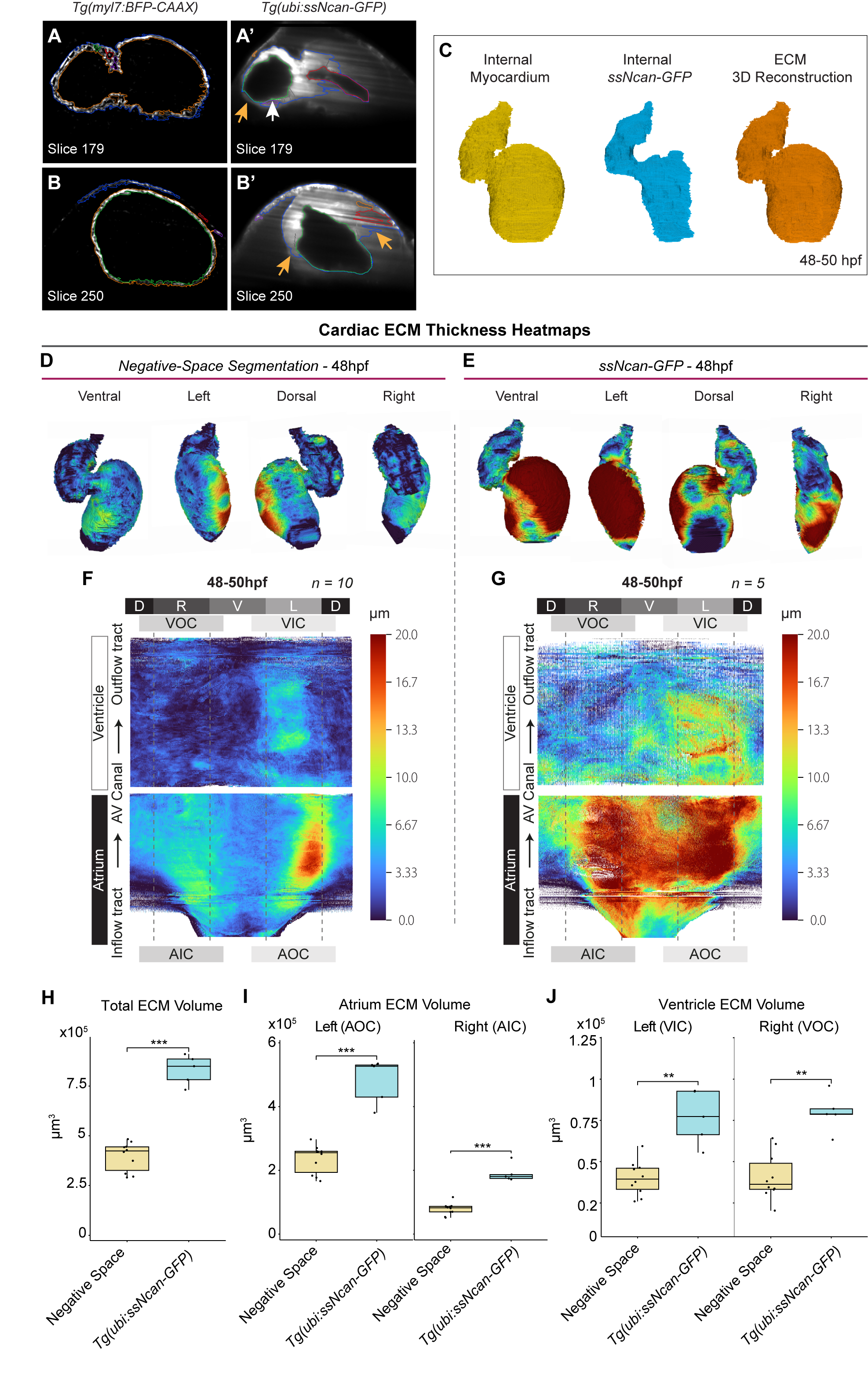

Supplement: S8 Fig — (A–B’) Example z-slices through Tg(myl7:BFP-CAAX) and Tg(ubi:ssNcan-EGFP) double transgenic hearts, highlighting the myocardium (A, B) and HA-biosensor (A’, B’), including contours automatically detected by morphoHeart. The myocardial signal shows tight and accurate contouring on both the outer and inner surface of the tissue, whereas the HA-biosensor signal around the heart is very variable, with variability in how tightly the internal ECM contour fits to the signal in the ventricle (white arrow), and failure to identify the outer contour of the cardiac ECM in the ventricle (yellow arrow). (C) 3D reconstructions of the internal myocardial mesh reconstruction (yellow), inner HA-biosensor mesh reconstruction (blue), and the resulting ECM mesh reconstruction (orange) generated by combining the first 2 meshes. (D, E) 3D heatmap reconstructions of cardiac ECM thickness in the negative-space ECM segmentation hearts (D) and the Tg(ubi:ssNcan-EGFP) hearts (E). (F, G) Average 2D unrolled ECM thickness heatmaps of negative-space ECM segmentation hearts (F) and Tg(ubi:ssNcan-EGFP) hearts (G). The cardiac ECM in Tg(ubi:ssNcan-EGFP) transgenic hearts appears generally thicker than that derived from negative-space segmentation, but shows regional distribution. (H–J) Comparative quantitative analysis between Tg(ubi:ssNcan-EGFP)-derived ECM and negative-space-derived ECM volumes in whole hearts (H), atria (I), and ventricles (J). In general, ECM volume is increased in the Tg(ubi:ssNcan-EGFP) HA-biosensor line compared to negative space segmentation, but the same patterns of ECM regionalisation (higher ECM volume in atrium compared to ventricle, higher ECM volume on atrial outer curvature) are observed. One-way ANOVA with multiple comparisons. ** p < 0.01, *** p < 0.001. Tg(ubi:ssNcan-GFP) 48 hpf n = 5; negative-space segmentation 48 hpf n = 10. Plots display median and quartiles. The numerical data underlying this figure can be found in S1 Data. (TIF) [file pbio.3002995.s008.tif]

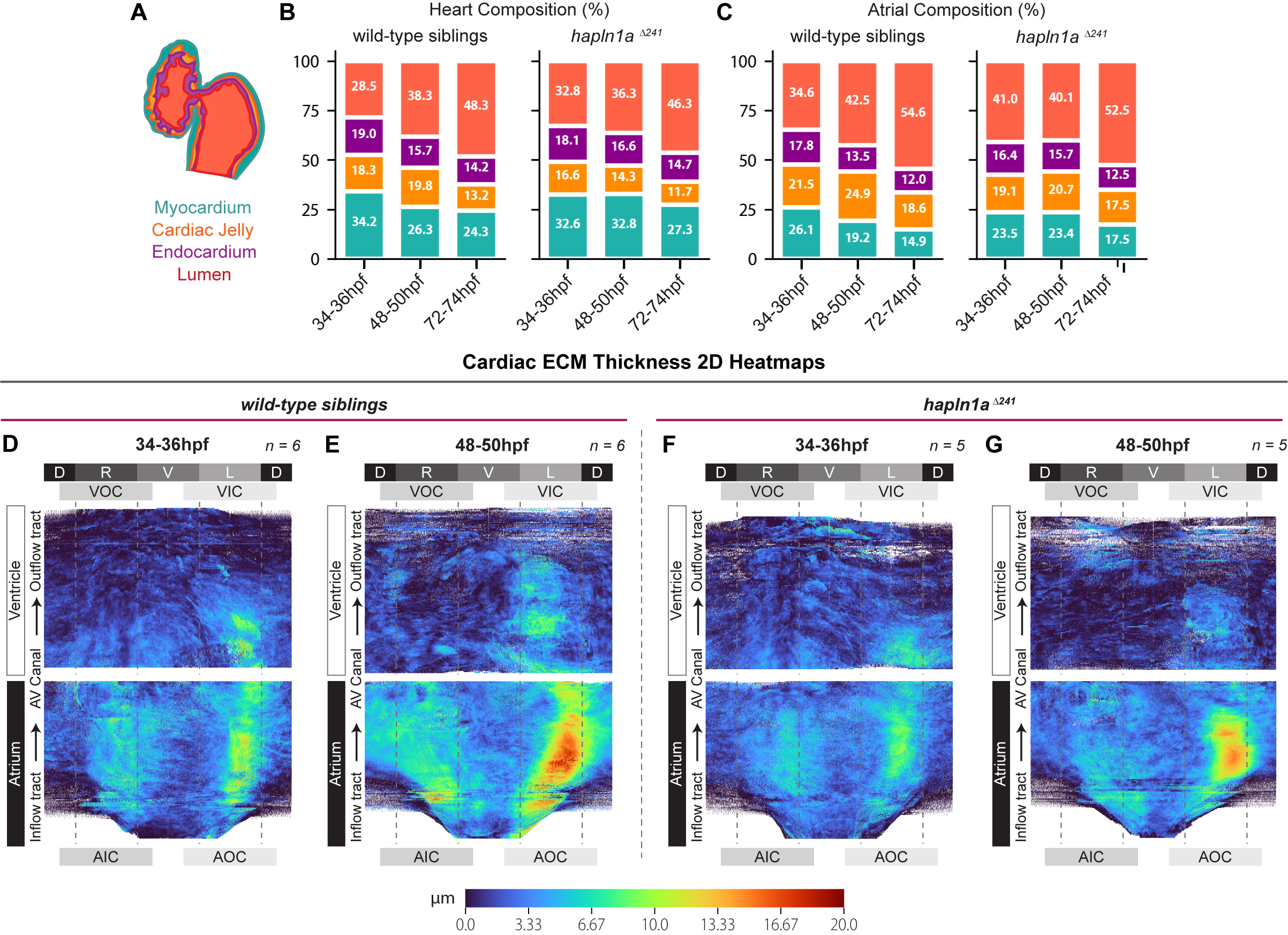

Supplement: S9 Fig — (A–C) Analysis of heart composition by percentage contribution of each compartment of the heart. Schematic depicts contributing tissues/regions of the heart (A). During early stages of heart morphogenesis, hapln1a mutants display a comparative reduction in contribution of ECM to total heart or atrial volume (B, C). (D–G) Averaged 2D ECM thickness heatmap reveals that while the magnitude of ECM expansion and regionalisation is reduced in hapln1a mutants at 34–36 hpf (F) and 48–50 hpf (G), a small region of the atrium still exhibits a thicker ECM. Labels around the heatmap indicate cardiac region: D—dorsal, V—ventral, L—left, R—right, AOC—atrial outer curvature, AIC—atrial inner curvature, VOC—ventricular outer curvature, VIC—ventricular inner curvature, AV Canal—atrioventricular canal. Plots display mean values for each category. The numerical data underlying this figure can be found in S1 Data. (TIF) [file pbio.3002995.s009.tif]
