# Supplementary material for: morphoHeart: A quantitative tool for integrated 3D morphometric analyses of heart and ECM during embryonic development
Source: PLoS Biol. 2025 Jan 29;23(1):e3002995. doi: 10.1371/journal.pbio.3002995 (PMC11778784; doi:10.1371/journal.pbio.3002995)
Supplement: S1 File — (DOCX) [file pbio.3002995.s010.docx]

**Pre-*morphoHeart* Masking and Cropping FIJI Macro**

| /*  **  merge_myoc_and_cj.ijm*  **  This macro will:*  **  - Ask the user to define a directory where the images are saved*  **  - Ask the user to open two individual channels of an image.*  **  - Create a MIP using both channels*  **  - Ask the user to create square in the MIP that includes all the ROI*  *(heart) and use such square to crop both channels*  **  - Ask the user to create a black square box to separate all signal from*  *both channels from the borders.*  **  Then, in each channel the macro will:*  **  - Enhance contrast using 0.3% saturated pixels and normalizing the*  *histogram*  **  - Enhance contrast equalizing the histogram*  **  - Despeckle (x3)*  **  - Save each cropped channel individually adding _EDC.tif to the filename*  **  Finally, for each channel the macro will create channel masks to use in*  *morphoHeart using the morphological filters from MorpholibJ*  ***  **  Created by:*  **  Juliana Sanchez-Posada*  ***  **/*  *macro "ECD and mask" {*  *print("\\Clear")*  *print("NEW RUN");*    *// Get images source directory*  *dirSource = getDirectory("Choose the directory where the final channels will be saved... ");*  *print("dirSource: "+dirSource);*  *run("ROI Manager...");*    *//Open channel 1*  *waitForUser("Open Channel 1 and click OK when ready.");*  *ch1_ID = getImageID();*  *ch1o_tt = getTitle();*  *ch1_tt = split(ch1o_tt, '.')*  *ch1_name = ch1_tt[0];*  *ch1_filename = dirSource + ch1_name;*  *print(ch1_ID, ch1o_tt, ch1_name);*  *print(ch1_filename);*    *//Open channel 2*  *waitForUser("Open Channel 2 and click OK when ready.");*  *ch2_ID = getImageID();*  *ch2o_tt = getTitle();*  *ch2_tt = split(ch2o_tt, '.');*  *ch2_name = ch2_tt[0];*  *ch2_filename = dirSource + ch2_name;*  *print(ch2_ID, ch2o_tt, ch2_name);*  *print(ch2_filename);*    *//Create EDC for both channels*  *// Ch1*  *selectImage(ch1o_tt);*  *fECandDx3();*  *ch1_EDC = getImageID();*  *ch1EDC_tt = getTitle();*    *// Ch2*  *selectImage(ch2o_tt);*  *fECandDx3();*  *ch2_EDC = getImageID();*  *ch2EDC_tt = getTitle();*  *//Merge channels*  *Label_Comp = "c2=["+ch2EDC_tt+"] c5=["+ch1EDC_tt+"] create keep";*  *run("Merge Channels...", Label_Comp);*  *//Create Max Intensity Projection*  *run("Z Project...", "projection=[Max Intensity]");*  *MAX_ID = getImageID();*  *MAX_tt = getTitle();*  *saveAs("tif", dirSource+ch1_name+"_"+ch2_name+"_EDC Composite");*    *// Set line width to 10*  *run("Line Width...", "line=10");*  *run("Colors...", "foreground=black background=black selection=yellow");*  *//Crop image*  *//Select MAX image to draw square to crop*  *selectImage(MAX_ID);*  *setTool("rectangle");*  *waitForUser("Draw square -Shift- that encompasses the whole heart and click OK when ready");*  *roiManager("Add");*    *//Crop MIP*  *selectImage(MAX_ID);*  *roiManager("Select", 0);*  *run("Crop");*  *saveAs("tif", dirSource+ch1_name+"_"+ch2_name+"_EDC Crop Composite");*    *//Crop ch1*  *selectImage(ch1_EDC);*  *roiManager("Select", 0);*  *run("Crop");*  *setTool("rectangle");*  *waitForUser("Draw rectangle that encloses the whole image and avoids open contours and click OK when ready");*  *if (selectionType() == 0) {*  *roiManager("Add");*  *run("Draw", "stack"); // same as Ctrl+D*  *print("Black rectangle drawn in ch1");*  *}*  *makeRectangle(0, 0, 1, 1);*  *saveAs("tif", ch1_filename+"_EDC");*  *ch1ECD_ID = getImageID();*  *ch1ECD_tt = getTitle();*    *//Crop ch2*  *selectImage(ch2_EDC);*  *roiManager("Select", 0);*  *run("Crop");*  *roiManager("Select", 1);*  *run("Draw", "stack"); // same as Ctrl+D*  *print("Black rectangle drawn in ch2");*  *makeRectangle(0, 0, 1, 1);*  *saveAs("tif", ch2_filename+"_EDC");*  *ch2ECD_ID = getImageID();*  *ch2ECD_tt = getTitle();*  *// Ask user to check the resulting cropped stacks and save them*  *waitForUser("Check stacks and click OK when ready to continue");*  *selectImage(ch1ECD_ID);*  *masking();*  *ch1mask_ID = getImageID();*  *ch1mask_tt = getTitle();*  *saveAs("tif", ch1_filename+"_mask");*  *selectImage(ch2ECD_ID);*  *masking();*  *ch2mask_ID = getImageID();*  *ch2mask_tt = getTitle();*  *saveAs("tif", ch2_filename+"_mask");*    *waitForUser("Check stacks and click OK when ready to close");*  *close("*");*  *roiManager("reset")*  *close("\\Others");*    *print("Images have been closed");*  *print("DONE: merge_myoc_and_cj");*  *}*  *function fECandDx3() {*  *run("Enhance Contrast...", "saturated=0.3 normalize process_all");*  *run("Despeckle", "stack");*  *run("Despeckle", "stack");*  *run("Despeckle", "stack");*  *}*  *function masking() {*  *run("Morphological Filters (3D)", "operation=Laplacian element=Cube x-radius=2 y-radius=2 z-radius=2");*  *run("Invert", "stack");*  *run("Threshold...");*  *setAutoThreshold("Li dark");*  *waitForUser("Set threshold (DO NOT CLICK APPLY) and click OK when ready");*  *run("Convert to Mask", "method=Li background=Dark black");*  *run("Invert", "stack");*  *run("Morphological Filters (3D)", "operation=Dilation element=Cube x-radius=1 y-radius=1 z-radius=1");*  *}* |
| --- |
|  |
