# Supplementary material for: morphoHeart: A quantitative tool for integrated 3D morphometric analyses of heart and ECM during embryonic development
Source: PLoS Biol. 2025 Jan 29;23(1):e3002995. doi: 10.1371/journal.pbio.3002995 (PMC11778784; doi:10.1371/journal.pbio.3002995)
Supplement: Attachment — Submitted filename: Sanchez Posada et al morphoHeart 2024 Response to Reviewers.docx [file pbio.3002995.s012.docx]

**Response to reviewers**

### Reviewer #1:

Morphogenesis is an incredibly complex and dynamic process. The ability to accurately and comprehensively report morphological findings has been hampered by the dearth of open-source, 3D tools that are navigable for investigators without extensive programming ability. With a rich history in cardiac developmental biology, Sáchez-Posada and Noël were well-positioned to understand the needs of the field, and, in this manuscript, the authors present a newly developed program, morphoHeart, that will aid researchers in understanding both wild-type morphology and specific deviations from it in various contexts. With the extensive resources associated with this manuscript and what appears to be a very user-friendly interface, this reviewer foresees the potential for wide application of morphoHeart. Minor additions, clarifications, and explicit instructions for the potential user may increase the uptake of morphoHeart in the developmental biology community, and beyond.

Indeed, the product itself appears quite complete in its usefulness; with this in mind, this reviewer supplies suggestions for manuscript revision that may boost the uptake of this software in the community.

We thank the reviewer for their positive appraisal of our software and manuscript, along with their comments and suggestions for improving our work, as well as the functionality for the user. Please find our detailed response to specific comments below.

1. Although the authors provide well-reasoned arguments for using live tissue as input for their particular experimental questions, there will certainly be scenarios where researchers need or want to use fixed tissue. It would be helpful for these readers to see a brief comparison between fixed and lived tissue, even if this is a simple comparison, such as some volume comparisons at one developmental stage. If such data aren't able to be included, it would be helpful to include at least a section in the Discussion that would inform users of anything to keep in mind if trying to use fixed tissue.

We agree with the reviewer that an illustration of the caveats of using fixed tissue would be beneficial for researchers who require fixed tissue analysis, to help understand/caveat their data. We have performed *morphoHeart* analysis of the myocardium in fixed embryos of the same transgenic line used throughout the bulk of the manuscript (*Tg(myl7:lifeActGFP))*. We find that in general volumetric measurements are reduced indicating significant shrinking of the tissue, and folding of the atrial myocardium can be observed, likely as a result of a thin atrial myocardium. We also find that some morphological measurements such as looping ratio are not affected, suggesting that fixed tissue analysis may still facilitate some accurate morphological analysis. We have included this data in Fig S6, and include the comparisons between live and fixed tissue in the Results section of the manuscript.

2. The ability to quantify "negative space" is indeed an interesting and attractive aspect of this program. However, others interested in the ECM itself may benefit from a brief discussion of the usage of actual ECM markers as input for morphoHeart. Have the authors tried this approach and compared it to the quantification of empty space? Are there certain scenarios where one might choose the ECM marker approach over the empty space approach?

We agree with the reviewer that the comparison of methods to segment the ECM is an attractive prospect, and therefore we set out to use this line to segment the cardiac ECM at 48-50hpf, comparing these findings with our negative-space ECM segmentation of the same stage. As predicted, we encountered difficulties in the automated thresholding of the *Tg(ubi:ssNcan-EGFP)* HA-sensor transgenic line, due to quite profound differences in intensity of the signal in different regions of the heart, expression in the pericardium, and background in the pericardial cavity (illustrated in panels in Fig S8). To overcome this issue, and allow us to segment the ECM as cleanly as possible using this transgenic, we took the following approach. We applied a threshold level to the contour-finding module in *morphoHeart* that allowed a relatively close segmentation of as much of the inner contour of the *Tg(ubi:ssNcan-EGFP)* signal as possible. This approach required manual contouring of a significant number of z-planes to ensure that the inner ECM contour in the ventricle was accurate, and that parts of the inner ECM contour in the atrium were not missing. This approach does not allow accurate segmentation of the outer ECM contour, as the signal is often less bright, particularly in the atrium. Therefore, the ‘outer’ ECM contour was defined as the inner myocardial contour, and it was the combination of these two contours (inner *Tg(myl7:BFP-CAAX)*, and inner *Tg(ubi:ssNcan-EGFP)*) that were used to reconstruct the ECM (Fig S8). We find in general that the ECM appears much thicker using this method than through negative space segmentation (see thickness heatmaps and quantifications in Fig S8), however the regionalisation of the ECM (thicker ECM in atrium compared to ventricle, thicker ECM on atrial outer curvature vs inner) is still present, demonstrating that negative-space segmentation analysis of ECM dynamics appears accurate. There are a number of hypotheses for why we see these differences in total ECM volume: 1) Not contouring tightly to the ssNcan-GFP signal and/or the myocardium/endocardium signal used for negative space segmentation, which may result in overestimation of ECM volume using the HA-sensor vs underestimation using the negative-space segmentation approach; 2) the ssNcan-GFP transgenic line causes steric expansion of the ECM. This may be possible as the biosensor works by overexpressing a GFP tag which is bound to the HA-binding domain of Neurocan - meaning that the HA in the ECM is bound to extra components in the matrix which may affect organisation, and we argue that label-free volumetric measurements may be more accurate than non-native biosensors. We have included this extra data and supplemental figure in the Results section, and have also made a new ‘Practical considerations for transgenic analysis’ section of the Discussion for this, and other new transgenic data.

3. The reviewer appreciates the authors' morphoHeart analysis of the hapln1a mutant as a comparison to previous methods. However, it seems this comparison could be even more useful with a few additions:

a. It would be interesting to see what the hapln1a ECM thickness heatmap would look like at 34-36 hpf, particularly given the early ECM phenotype shown in Derrick et al. (2022) and the difference in atrial ECM volume at 34-36 hpf in Fig. 7K.

We have now added both 34-36hpf and 48-50hpf 2D ECM heatmaps from wild type and *hapln1a* mutants in Figure S9, as we agree that this allows a better comparison of the data generated by *morphoHeart* with that from our previous publication. Similar to the data at 48-50hpf, the 34-36hpf 2D ECM heatmaps demonstrate that in general the atrial ECM thickness is reduced in the *hapln1a* mutants, but that a similar small region of ECM thickness remains, that we had not been able to detect using our previous cross sectional analysis in Derrick et al 2022. We have included an extra sentence in the discussion that covers this comparison and highlights the power of *morphoHeart* for more detailed analyses of ECM regionalisation.

b. It seems that morphoHeart will be particularly useful as a "phenotype finder" for a new or relatively uncharacterized mutant, and one can imagine that the regional expansion heatmaps (as in Fig. 3) would be especially informative to help one hone in on a specific region of an organ for further study. The authors use several of the tests in their arsenal to explain the dysmorphia of the hapln1a heart, but it seems a missed opportunity not to show a comparison to wild-type regional expansion at 50 hpf.

We have now included 2D ballooning heatmaps for wild type embryos and *hapln1a* mutants at 48-50hpf in Figure 7, which demonstrates the failure in atrial chamber expansion specifically.

c. Given the parallels that the authors can now make between their current and previous analyses of hapln1a mutants, a discussion of the pros and cons of these approaches would be satisfying. For example, what can this new method tell us about the nature of the ECM in hapln1a mutants that couldn't be seen from the types of analyses in Derrick et al. (2022)? Ultimately, in what scenarios do the authors suggest that investigators choose one approach over the other — or is there room and necessity for both?

In our previous study, we performed a relatively rudimentary analysis of ECM regionalisation - using optical reslicing of the heart tube to measure left and right ECM thickness along the heart tube and in looped hearts. Using that analysis, we were able to identify a significant reduction in left-sided ECM expansion in the *hapln1a* mutant at 26hpf, and indications of a reduction in left-sided expansion at 48hpf. For the latter analysis we had constructed rose plots to visualise ECM distribution, but this type of analysis did not allow us to perform statistical testing, and was subject to high levels of variation due to difficulties in standardising the positions of the cross sections in each chamber in looped hearts.

The overall advantages of *morphoHeart* for ECM analysis are already detailed in the discussion, so we focus here on the additional information we gain about the *hapln1a* mutant phenotype compared to our previous publication: a) Hapln1a drives the atrial ECM expansion that occurs between 1dpf and 2dpf, something we could not previously quantify; b) contrary to our previous hypothesis, Hapln1a appears to be not the only factor driving regionalised expansion of the ECM since we can now appreciate that a small region of thicker ECM remains in the atrium of *hapln1a* mutants; c) Hapln1a is required, directly or indirectly, for the more modest ECM regionalisation in the ventricle; d) Hapln1a-drive ECM expansion between 1dpf and 2dpf promotes expansion of the atrial lumen

Regarding the reviewers second point here - we would consider *morphoHeart* to be the better method for analysing ECM regionalisation, as it allows a deeper analysis of ECM distribution, proper quantitation, and contextualisation of the ECM phenotype in relation to other heart and tissue layer morphologies. However, the main pinch-point is time - depending on how clean the input images are, negative-space segmentation can be time-consuming, and this is likely where the cost-benefit analysis should lie.

We have included some of these points regarding *hapln1a* in the ‘Cardiac ECM’ section of the Discussion, and a brief outline of time considerations in the ‘Limitations’ section.

4. Of course, it's not reasonable for the authors to discuss every possible type of experiment for which users might employ morphoHeart/morphoCell in the future. However, it may be worthwhile for the authors to provide their vision for the categories of experiments that are possible now or with some modification of the software, and what categories are likely out of bounds. For example, could they comment on the feasibility of quantifying the percentage of myocardial volume occupied by Golgi, or the proximity of adherens junctions to a particular contour?

We have expanded the *‘morphoHeart*: a novel tool….’ section of the Discussion to include a paragraph considering different applications of morphoHeart (where it could be ‘ready to go’ in its current form, or where it may need to be modified. For example, analysis of other organs such as the developing ear could yield new insights through using *morphoHeart*, as negative space segmentation of for example the lumen of the developing ear, along with epithelial morphology of the tissue, would allow a better understanding of how the tissue changes shape and organisation at it develops. Other suggestions, such as proximity of junctions to contours, would likely require the researcher to develop an add-on module using some of the visualisation and measuring functions of *morphoHeart*. *morphoHeart* is an open-source tool, and all the code is freely available for users to adapt as they wish.

Clarifications on software methodology:

1. Line 127: The sentence "To extract the myocardium and endocardium, individual slices making up each channel go through a process of contour detection and selection, extracting the contours that delineate the tissue layer." implies that contour identification is automatic, but explicit inclusion of this fact would be edifying to potential users. Additionally, this may be a good place to include some instruction on what makes a "good" marker for morphoHeart (perhaps a briefer version of the "Transgenic Line Selection" from the User Manual).

We have clarified in the text that contour identification is automatic, and contour selection is semi-automatic (i.e supervised) (new line 136+). We have also included a new section in the Discussion on ‘Practical considerations for transgenic line selection’, which together with the Discussion above on the different transgenic lines we segment in the paper itself, contains also the following text: ‘*To obtain the most accurate data using morphoHeart, the transgenic line and image acquisition approach need to be carefully evaluated and selected according to the biological question of interest. Some considerations to keep in mind are fluorophore selection and localization. As the cell morphology of each heart tissue is different (i.e. myocardial cells have uniform thickness and endocardial cells are thicker around the nucleus and thinner at junctions (Bornhorst et al., 2019; Schlaeppi et al., 2021)), and the cellular arrangement within the tissue changes as the heart develops (i.e. monolayer, multilayer), some transgenic lines are better for studying heart morphology and tissue distribution using morphoHeart. Accordingly, to segment the endocardium, lines that label cell membranes are recommended to avoid 'holes' in regions where the signal emission is very low due to thin cytoplasm. Bright, stable fluorophores are advised for deep heart imaging of the myocardium and long time-lapse studies to maintain image quality and prevent photo-bleaching and phototoxicity.’*

2. Readers may wonder about the projection method used to unroll the heatmaps, particularly because there seems to be missing signal in the VIC in Fig. 3M-O, for example. The reviewer suggests a brief discussion of the process and the potential for associated issues when introducing the first usage of this projection method.

The gaps in the 2D heatmaps corresponding to the VIC is a function of mapping 3D heart geometry into a 2D matrix. This works by: 1) dividing the centreline into an equally-distanced number of points; 2) making a transverse section through the heart at each of these points (see Fig S4A-B); 3) labelling each of the heart’s points in each transverse section with a radial value between -180° and 180°; 4), taking the mesh thickness/expansion value for the relevant heatmap at each point; 5) creating a 2D projected representation of the 3D heatmap by plotting in a colour-coded way the heatmap values for each radial position (in 𝓍) against the plane number (in 𝑦).

As the heart becomes more looped, the inner curvature has significantly less ‘points’ on its mesh than the outer curvature, and morphoHeart will not use the same mesh point more than once when creating all the transverse planes. This results in some transverse sections not having a value on the shortest part of the chamber - i.e the inner curvatures. This tissue or signal is therefore not missing per se. We do not extrapolate or duplicate values into these ‘gaps’ as this would constitute adding tissue that doesn’t exist.

Potential caveats to such an approach could be since the positioning of this gap is slightly different between organs (as looping morphology is rarely identical), while the number of embryos that contributes to an averaged heatmap can be high, the number of points that contribute to the averaged inner curvature values may be less, as some hearts will have values to contribute, and others will not. At present, without the use of e.g. adaptive resolution based on local geometry, we do not have a solution to this issue, and encourage researchers to always visualise both 3D and 2D heatmaps. We have included this latter consideration into a new Discussion section *‘Practical considerations for analysis of 2D heatmaps*’ as well as in the Limitations section of the manuscript.

3. It is somewhat unclear at what point and in which situations samples are "averaged" into a stereotypical organ. A brief discussion on this point should help readers to understand the types of analyses for which they can expect to be able to visualize biological variation (e.g. ECM volume), and when this is not possible (e.g. ECM heatmaps). Along these lines, what would be best practices for analysis of a mutant phenotype that includes a lot of morphological variation between individuals (so as to avoid missing key elements of the phenotype)?

To clarify in the figures which heatmaps represent averages, and which represent individual samples, we have included numbers of embryos directly in the figures next to those heatmaps which represent averages, and have clarified this also in the figure legends in places where we had not stipulated that 2D heatmaps were averaged.

Regarding best practice for analysing mutant phenotypes in particular where morphological variation may exist: in general we recommend that researchers visualise both 3D individual and 2D averaged heatmaps to identify both well-conserved phenotypes, and phenotypes that may be more variable in terms of expressivity, or phenotypes that are consistently observed, but perhaps stochastic in positioning. Quantitative analyses for certain parameters (for example volumetric measurements), should be used as an accessory method for visualising both overall phenotype and variability between samples.

One analysis that could be performed using *morphoHeart* 2D heatmap data is investigating and visualising variation. The 2D matrices that are used to construct each heatmap can be exported as .csv files. This allows the researcher to calculate standard deviation for every position in the 2D matrix, providing statistical measurement of variability across the tissue, which could in theory be represented as a 2D phenotypic variability heatmap. This ability to export the .csv files containing the 2D heatmap matrix information will also allow researchers to ‘divide’ the heart up into regions, average values across regions (for example average myocardial ballooning in the atrial outer curvature vs the inner curvature), and perform statistical analysis of how thickness or ballooning measurements differ between regions, across stages, or between genotypes.

We have included some of these considerations in the *‘‘Practical considerations for analysis of 2D heatmaps*’ section of the Discussion.

Additional instructions for usage:

1. What are the exact system requirements for the software to run? Currently, the User Manual states "morphoHeart currently works only in Windows", implying a fairly rudimentary system is acceptable. However, an explicit statement of this would make the software especially attractive to potential users (or in contrast, if a more sophisticated system is necessary, this information is especially important). Because this may change from version to version, and on different platforms (for example, when morphoHeart becomes available for other operating systems), the User Manual is a perfectly acceptable place for this information.

We have successfully tested *morphoHeart* on a range of PCs with varying specs. We have now included minimum and recommended system requirements in the User Manual.

2. Some more guidance from the authors on experimental setup may make potential users more comfortable. For example: Are other imaging setups acceptable (i.e. confocal instead of lightsheet)? What is the optimal step size between slices? Is there an upper limit to number of slices (or total file size) imported into the software?

To facilitate this for the user, we have included a brief sentence in the ‘morphoHeart: a new tool…..’ section of the Discussion that assures readers that any imaging modality is suitable as long as the image stacks are clean enough (i.e. good signal-to-noise) and saved as TIF files. We have expanded slightly the user manual to address some of the other technical considerations.

### Reviewer #2:

The manuscript by Possada and Noel presents morphoHeart, a new computational tool that allows for quantitative analysis of heart morphology in 3D. Using zebrafish as a model, the authors use this newly developed software to track heart development between 34 and 74 hours post fertilization (hpf). morphoHeart extracts external and internal contours of the two different layers of the heart, i.e. the myocardium (labeled with Tg(myl7:lifeact-GFP) and the endocardium (Tg(fli1a:TagRFP)), allowing for volumetric and geometric quantification of the cardiac tissue and the lumen. It can calculate the centerline of the heart, which allows for assessment of angular evolution during the looping process (looping ratio) and of tissue thickness maps in 2D. The authors quantified the extracellular space/ECM between the two tissues by quantifying the negative space between them. Through morphoHeart, the authors can distinguish regional differences in the heart at the tissue and the ECM levels, not only between atrium and ventricle, but also between inner and outer curvature of the heart.

Overall, the authors presented a very nice tool that would be useful for scientists working in the field of morphogenesis. The analysis appears strongly quantified and detailed, with regional distinctions that have not been clearly reported in previous publications. The software also appears to be generalizable to many different 3D systems with well-established fluorescent tools/markers. Currently, the manuscript mostly focuses on descriptive features of the developing heart, except for differences between wild types and hapln1a mutants. However, future work from the authors and others in the field would benefit from this software in analyzing different genetic conditions and loss-of-function phenotypes.

We thank the reviewer for their positive appraisal of our work, along with their comments and suggestions for improving the manuscript. Please find our detailed response to specific comments below.

Major comments

-	The sample sizes are not very big (in most cases, n is 10 or lower), which might account for the high variability of the values particularly in 72-74 hpf animals. The data might also not necessarily have a normal distribution due to the limited sample size. It would be better evidence if the authors could expand their numbers.

We have expanded the number of samples analysed at 72-74hpf to bring it in line with the other stages (n=10). This expansion has not changed any of the main findings in the paper (although some morphometric changes at 72-74hpf have become more significant). n=10 is not a particularly low number of samples to analyse for live zebrafish embryology or mouse embryonic heart phenotypic analysis. Furthermore, we are able to identify significant morphological phenotypes when comparing wild type embryos to *hapln1a* mutants, with n=5, demonstrating the power of *morphoHeart* to identify morphological defects with lower sample sizes than n=10. We think one of the advantages of using software such as morphoHeart is that it enables a broader and deeper analysis of morphology that is more powerful than other approaches, reducing the number of replicates required for analysis.

In particular, the authors could use a different combination of transgenic animals than the one already used in the paper (Tg(myl7:lifeact-GFP), Tg(fli1a:TagRFP)), e.g. with a red myocardial marker and a green endocardial marker, to assess the consistency of their results. This would also alleviate any concerns of non-specific effects, in particular because lifeact-GFP could exert side effects for cell morphology and development, and confirm the specificity of their results. The authors have also already performed imaging with Tg(myl7:BFP-CAAX); were the results of myocardial volume measurements consistent with what was observed with Tg(myl7:lifeact-GFP)?

We agree with the reviewer that it is possible that the transgenic line used could impact the outputs from *morphoHeart*, for several reasons (difference in expansiveness of labelling within individuals cells/total tissue, background-specific effects related to transgenic line/insertion site etc). Indeed, in our response to reviewer 1, where we segment the ECM using the *Tg(ubi:ssNcan-EGFP)* transgenic HA-sensor line, we see differences in overall ECM volumes, although importantly the regionalisation of the ECM we observe through negative-space segmentation is preserved (see response to reviewer 1, and new Fig S8).

To investigate this phenomenon further we therefore performed segmentation of two extra myocardial transgenic lines: the published *Tg(myl7:BFP-CAAX)* line, in which myocardial membranes are labelled with BFP, and a new *Tg(myl7:Citrine)* line we generated in our lab, in which a non-localised Citrine is expressed in cardiomyocytes (note this line was made by Dr Christopher Derrick, who has been added as an author on the manuscript). This data is presented in new Fig S5.

When comparing *Tg(myl7:BFP-CAAX)* hearts with *Tg(myl7:lifeActGFP)* hearts at 48-50hpf, we observe some differences. The ventricles appear smaller (evident through both quantitative analysis, and ballooning heatmap analysis), which does not appear to be driven by a reduction in myocardial volume, but rather less expansion of the chamber. We also observe that while the atria are the same size in general between the two lines, there is less myocardial tissue in *Tg(myl7:BFP-CAAX)* atria. Analysis of myocardial thickness 3D heatmaps demonstrates that this is due to poor signal in the dorsal (back) face of the atrium, and thus some ‘loss’ of atrial tissue when segmenting the myocardial tissue using this specific transgenic line, when compared to the *Tg(myl7:lifeActGFP)* line. Looping ratio is unaffected, suggesting looping itself is unaffected, but also revealing the power of *morphoHeart* to identify small conserved differences in heart morphology or growth between populations.

When comparing *Tg(myl7:Citrine)* hearts with *Tg(myl7:lifeActGFP)* hearts at 72-74hpf, we again observe some differences in volumes, although not the same as those observed in the *Tg(myl7:BFP-CAAX)* line. We find that overall atrial size is increased in *Tg(myl7:Citrine)* transgenics compared to the *Tg(myl7:lifeActGFP)* line, while ventricular size is reduced. This increase in atrial size is due to a more advanced growth of the chamber (as evidence by greater chamber ballooning and coincident greater atrial myocardial volume), whereas the reduced ventricular size appears to be the result of less ballooning of the ventricle. Again, similar to our finding with the *Tg(myl7:BFP-CAAX)* line, looping ratio is not affected, and neither were pole-to-pole measurements.

We therefore do acknowledge that morphological parameters may vary depending on the transgenic line used, but the mechanism underlying this will be harder to understand. Due to the nature of the differences we see (particularly for total chamber volume/elevated or reduced ballooning of the chamber), we speculate this represents subtle differences in baseline morphology or developmental timing due to different backgrounds of zebrafish lines, or insertional effects of individual transgenes. Our recommendations are therefore that in studies comparing organ dynamics over time, researchers are consistent in their use of transgenic lines at different stages to avoid introducing any line-specific confounding factors.

We have included a summarisation of the data and related issues in the Practical considerations for analysis of transgenic lines’ section of the Discussion.

-	Depending on the angle of the embryo mounting and depth of tissue, fluorescence signal intensity can be difficult to detect throughout the whole heart. Have the authors accounted for thorough detection to account for the entire volume of the heart or the lumen, which might result in detection of atrial volume reduction at 74 hpf? Including representative images of the z-stack or a 3D reconstruction of the heart in Supplemental figures would be instructive.

We agree that the combination of transgenic signal and embryo mounting can influence the strength of the fluorescent signal detected in our 3D images, and that loss of this signal in deeper regions of the heart could affect reconstructions (which would be reflected not only in the volumetric readings, but also in e.g. the thickness and ballooning heatmaps). We are careful to select samples to segment in which we are confident that we have captured signal around the whole chamber for both ventricle and atrium (with the obvious exception of the openings that occur at the inflow and outflow). We have included some representative z-slices distributed throughout a 72-74hpf heart in Figure S1 as illustration of the kind of input we have used, as well as snapshots from a standard 3D reconstruction of the processed pre-morphoHeart image data and its corresponding 3D segmentation obtained with *morphoHeart*, both showing no loss of signal at the dorsal surface.

-	Considering that morphoCell was performed on "Tg(myl7:BFP-CAAX); Tg(myl7:H2B-mScarlet", did the authors assess whether the individual sizes/volumes of CMs can be measured using the membrane marker instead of the inter-nuclear distance or myocardial wall thickness? If not, what are the limitations of the software that prevent the use of the BFP signal?

In theory, morphoHeart can be used to segment cell volume using a membrane marker. However in order to do so, the user would need to do the following:

1) Image at sufficient resolution that membranes are clear and distinguishable. Since we used the myl7:BFP-CAAX line to visualise at tissue resolution (rather than focussing on imaging parameters that ensure individual membranes were distinguishable across the whole heart), our images were not suitable for integration into *morphoHeart* with the intention of segmenting individual cells

2) In *morphoHeart’*s current form, to measure the volume of each cell they would need to be segmented as individual channels or tissues. Therefore users would need to input the number of cells they wish to segment/analyse volume of, giving each of them individual tissue/cell identifiers in the Project Setup page. This would allow the capture of parameters of individual cells. This is unlikely to be practical for analysis of cell volume across the whole heart, but could be used to analyse e.g. a small number of cells in specific regions of the heart. A similar approach could be used for e.g. random mosaic labelling of cells, where each mosaic cell could be an individual identifier, and its volume and geometry analysed in isolation.

-	The finding that the ventricle ECM significantly expands only in a very limited time window (between 34 and 48 hpf) and immediately decreases at 56 hpf is surprising. Mechanistically, how do the authors speculate this occurs (purely by degradation?) and what would be the functional consequence were this expansion to be abolished? Do the authors have a model in which this ECM expansion between 34 and 48 hpf is abolished?

We speculate that ECM volume increasing and decreasing in the time frame outlined in our papers may be related to a shift from the ‘morphogenetic’ phase (i.e. a more HA-rich osmotically swollen ECM which may be softer and more pliable for tissue deformation) to the ‘functional’ phase (i.e. less interstitial matrix meaning a stiffer tissue that supports cardiac function) of heart development, and studies in chick embryos have shown that the heart tube does indeed stiffen as development progress (Majkut et al, 2013).

In this manuscript we present the *hapln1a* mutant in which ECM expansion between 34hpf and 50hpf is defective, not only in the atrium but to a lesser extent in the ventricle. We have previously found no impact in heart rate in these mutants, but have not assessed contractility or e.g. trabeculation dynamics in more detail. Since we have been the first to describe this expansion in ECM between 34hpf and 50hpf, we are not aware of other models in which this expansion is defective (but they could of course be out there, and we hope our tool facilitates analysis of this phenomenon).

Regarding the reduction in ECM volume - this could be due to several factors: reduction in ECM hydration (less osmotic swelling); active degradation or increased turnover of HA or other interstitial matrix components; changes in ECM synthesis. There is already evidence for the latter - for example Versican switches from being expressed throughout the heart at tube stage to restricted to the AV canal once the heart has looped at 48hpf, however this does not rule out a relative contribution of several mechanisms. These are mentioned briefly in the ‘ECM’ section of the discussion, and we are currently investigating some of these possibilities for future manuscripts.

-	Similarly, what do the authors speculate is the reason behind the reduction of atrial myocardial tissue volume after 48 hpf?

We hypothesise that the reduction in atrial tissue volume (and reduction in atrial CM size in some regions) may be related to the accompanying changes in ECM volume - one such mechanism that could link these is coherent osmotic regulation of ECM and cell volume, similar to the model that has been described in the atrioventricular valve (Vignes et al, 2022). However, we acknowledge that this hypothesis has yet to be tested.

-	The ECM regionalization appears to be primarily driven by cardiac trabeculation. What happens to the ECM volumes in the different regions in animals with no trabeculae? It would be interesting to test out morphoHeart in ErbB2-inhibited animals.

We agree with the reviewer that ECM regionalisation correlates well with the regionalisation of trabeculation (i.e ECM is thickest in regions where trabeculae will not form - thickest in atrium, less thick in inner curvature of ventricle, and thinnest in ventricle outer curvature), and that the relationship between these phenomena could be very interesting. However, we do not speculate that trabeculation drives this ECM regionalisation, (indeed, the regionalisation is present **before** the onset of ventricular cardiomyocyte delamination, rather than the other way round), and in this particular manuscript we do not wish to make strong statements about the functional nature of this relationship in either direction. This concept is being investigated further in collaboration with another group, with whom a manuscript is being prepared. Since this constitutes part of a larger collaborative story, we do not plan to include that data in this manuscript.

Minor comments

-	Specify what "GUI" stands for in the abstract.

We have updated this in the manuscript.

-	I would use a term other than "degradation", particularly when describing the hapln1a mutant phenotype, as there is no direct evidence yet that the ECM is actively being removed.

We have updated the text to read ‘reduction, degradation or compaction’ to reflect the fact that it is unclear what mechanism underlies the lower ECM volume in hapln1a mutants.

-	On page 14, "We reveal a novel requirement for in driving expansion of the atrial lumen", the word "Hapln1a" is missing.

We have updated the text accordingly

-	The abbreviation "SHF" needs to be clarified.

We have defined this abbreviation upon first mention in the text

-	Scale bars are missing from Figure 6F and 4G.

We have updated the figure panels accordingly.

-	Is 10^5 correct on the y axis of the graphs in Figure 7H-J?

We thank the reviewer for drawing our attention to this, have checked the y-axis units in the panels referencing myocardial tissue volume and they are indeed 10^5.

-	Figure Legend 3H-J should be H-K.

We have corrected this in the text

### Reviewer #3:

The manuscript by Sánchez-Posada and Noël aims to fill a gap in analysing early heart development, particularly heart morphogenesis in live embryos. Existing methods have limitations, like collapse or shrinkage of tissue due to fixed tissue, which can alter results. The authors propose morphoHeart as a tool to fill the current gap by offering a comprehensive pipeline for semi-automated segmentation and analysis of developing hearts, including segmentation of the cardiac ECM. The software's graphical user interface expands its utility beyond cardiac tissue, allowing analysis of multiple tissue layers and, importantly, negative space segmentation. The latter is one of the significant contributions of morphoHeart - its ability to provide negative space segmentation of the cardiac ECM, a feature not available in other analytic approaches. Additionally, it provides high resolution and methodical detail in quantifying cardiac tissues of developing zebrafish hearts.

The open-source nature of morphoHeart is a critical aspect of its contribution. It enables other researchers to expand its capabilities, implementing new quantifications or descriptors to support analysis of various tissues, making it attractive also to non-cardiac users. This open-access approach fosters collaboration and innovation in the field; therefore the authors should be commended by their effort.

The paper presents a detailed analysis of the zebrafish heart and uses morphoHeart to systematically characterise in 3D how the heart undergoes periods of growth and compaction during early morphogenesis, including quantifying distinct geometric changes during heart looping and chamber expansion, and the regionalised and chamber-specific ECM volumetric remodelling that happens during these stages of development. The inclusion of Hapln1a mutants in the manuscript as a way to support morphoHeart's capability in delivering more detailed analyses of mutant phenotypes is particularly welcomed.

Overall, morphoHeart represents a significant step forward in the analysis of early heart morphogenesis. Its potential adaptability to other systems and organs, along with its open-access nature, make it a valuable tool for the scientific community. There are only a few minor points that require attention, and these are described below:

We thank the reviewer for their positive appraisal of our paper, along with their comments and suggestions for improving the accessibility of our work to the community. Please find our detailed response to specific comments below.

Figure 1: please avoid using red and green colours in the same figure (Fig 1J,) for readability and inclusivity purposes.Thanks to the reviewer for pointing this out. In the schematic we have changed the green contour of the myocardium to a brighter green and the internal contour of the myocardium to a darker red. We have checked with an online tool (https://projects.susielu.com/viz-palette) how these colours might appear to populations with different colour deficiencies (for the specific colours chosen see: https://projects.susielu.com/viz-palette?colors=[%22#dc133b%22,%22#3ff44c%22]&backgroundColor=%22white%22&fontColor=%22black%22&mode=%22deuteranopia%22)

Figure 3: The labels around the 2D heatmaps which indicate cardiac region should be coloured in a different way (Fig 3 L-O). At the moment it is confusing to the non-specialised reader, as one could assume the green/orange/pink/blue colours could either associate with each other or even with the heatmap. Use a different set of colour pallet altogether.

We thank the reviewer for pointing this out and agree the extensive use of colours in the key could be confusing in combination with the colour scales used for the heatmaps. We have changed the regional labels around the 2D heatmaps to a greyscale palette, which we hope will avoid any confusion - and have amended all figures in which these 2D heatmaps appear, for consistency.

Figure 4: Please include scale bar in Fig 4J.

We have included a scale bar on Figure 4 panel F (we think this is the panel the reviewer is referring to).

Figure 5: Similarly, the labels around the 2D heatmaps which indicate cardiac region should be coloured in a different way (Fig 5 K-N). At the moment it is confusing to the non-specialised reader, as one could assume the green/orange/pink/blue colours could either associate with each other or even with the heatmap. Use a different set of colour pallet altogether. For Fig 5E, please avoid using red and green colours in the same figure for readability and inclusivity purposes.

Similar to Figure 3, we have updated the palette around the heatmaps to a greyscale palette, and in the schematic have changed the red and green colours used in the myocardial inner contour and tissue, respectively.

Figure 6: Similarly, the labels around the 2D heatmaps which indicate cardiac region should be coloured in a different way (Fig 6 L-O). At the moment it is confusing to the non-specialised reader, as one could assume the green/orange/pink/blue colours could either associate with each other or even with the heatmap. Use a different set of colour pallet altogether.

Similar to Figures 3 and 5 we have updated the palette around the heatmaps to a greyscale palette.

Figure 7: It is not clear how many WT and hapln1a mutant hearts have been imaged and analysed in this figure to be included in the analysis presented in panels C-M. Can the authors please include this information in the figure legend, as they have done for the other figure legends?

We have now included the number of embryos analysed for each stage and genotype in the figure and legend.
